# m6A modification on the fate of colorectal cancer: functions and mechanisms of cell proliferation and tumorigenesis

**DOI:** 10.3389/fonc.2023.1162300

**Published:** 2023-04-21

**Authors:** Xiaohan Jiang, Ziyao Jin, Yuzhong Yang, Xiang Zheng, Shaohua Chen, Shuaijie Wang, Xuemei Zhang, Nanfang Qu

**Affiliations:** ^1^ Department of Pathology, Affiliated Hospital of Guilin Medical University, Guilin, China; ^2^ Key Laboratory of Oral Biomedical Research of Zhejiang Province, Stomatology Hospital, School of Stomatology, Zhejiang University School of Medicine, Hangzhou, China; ^3^ Department of Breast and Thyroid Surgery, Liuzhou People’s Hospital Affiliated to Guangxi Medical University, Liuzhou, China; ^4^ Department of Pathology, Liuzhou People’s Hospital Affiliated to Guangxi Medical University, Liuzhou, China; ^5^ Department of Gastroenterology, Affiliated Hospital of Guilin Medical University, Guilin, China

**Keywords:** colorectal cancer, N6-methyladenosine, RNA modification, proliferation, tumorigenesis

## Abstract

N6-methyladenosine (m6A) is the most pervasive RNA modification in eukaryotic cells. The dynamic and reversible m6A modification of RNA plays a critical role in the occurrence and progression of tumors by regulating RNA metabolism, including translocation, mRNA stability or decay, pre-mRNA splicing, and lncRNA processing. Numerous studies have shown that m6A modification is involved in the development of various cancers. This review aims to summarize the significant role of m6A modification in the proliferation and tumorigenesis of CRC, as well as the potential of modulating m6A modification for tumor treatment. These findings may offer new therapeutic strategies for clinical implementation of m6A modification in CRC in the near future.

## 1 Introduction

Colorectal cancer (CRC) is a common malignant tumor that occurs in the gastrointestinal tract, and its mortality rate ranks fourth among global malignant tumors (1). More concerning is the rising incidence in individuals younger than 50 years old and the surge in CRC incidence among young adults (2, 3). Genetic and environmental factors, lifestyle, and obesity are all critical in the pathogenesis of colon cancer. A vast body of scientific evidence unequivocally demonstrated that the post-transcriptional RNA modification, N6-methyladenosine (m6A), has a central role in the pathogenesis of CRC (4–6).

**Figure 1:**
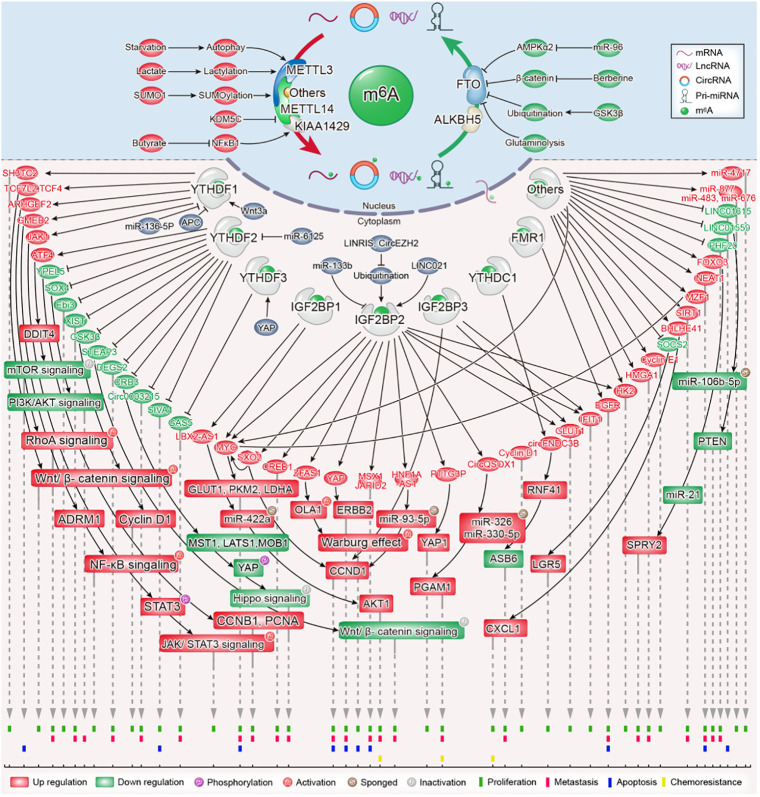
The role and molecular mechanism of m6A modification in colorectal cancer.

m6A refers to the methylation of adenosine at the N6 position. It is the most abundant internal messenger RNA (mRNA) modification in eukaryotes and was first identified in mammalian mRNA in the 1970s (7–9). Noncoding RNAs, such as tRNAs and rRNAs, are also modified and depend on these modifications for their biogenesis and function (10–12). As a conservative RNA modification, m6A exists in most tissues, such as viruses, yeast, plants, and mammals (13–17). This modification is reversible and dynamic, regulated by methyltransferases (also called “writers”), demethylases (also called “erasers”), and methylation-binding proteins (also called “readers”). RNA can be methylated and demethylated by dedicated “writers” and “erasers,” respectively. The multifunctional role of m6A on RNA fate decisions is determined by “readers.” It is now evident that the recognition of m6A modification is carried out by selectively binding reader proteins, which affects the translation efficiency, stability, decay, and lifetime of RNA (18–20). The functional significance of m6A modifications has been reported in a variety of diseases, including digestive tract carcinomas (4, 5). Researchers have identified that sustaining proliferative signaling and evading growth suppressors as the hallmarks of cancer (21). Here, we provide an up-to-date review of the role of m6A modification in cancer cell proliferation and tumorigenesis within CRC (**Figure 1**). Additionally, we emphasize the potential clinical applications of m6A modification as a major focus of this review.

## 2 The functional roles of m6A “writers” in CRC proliferation and tumorigenesis

The m6A methyltransferase complex consists of multiple “writer” protein components. METTL3 and METT14 are the core m6A “writers,” both of which have methyltransferase domains. METTL3 has a catalytic effect, whereas METTL14 does not. The primary function of METTL14 is to act as an RNA-binding platform that provides structural support for METTL3 and recognizes RNA substrates (22, 23). WTAP acts as a regulatory subunit of the m6A methyltransferase complex; it interacts with METTL3/METTL14 heterodimers and has no methyltransferase capacity (24). However, it is indispensable for the nuclear speckle localization and catalytic activity of METTL3/METTL14 heterodimers (25). Other well-known “writer” proteins include KIAA1429 (VIRMA), METTL16, RBM15/15B, and ZC3H13 (26, 27). The latest studies have provided a better understanding of the role of METTL3 and METTL14 in the cell proliferation and tumorigenesis of CRC; therefore, we perform a comprehensive analysis on these two writer proteins (**Table 1**).

**Table 1:** Functions of m6A “writers” in CRC.

Gene Symbol	Expression in CRC	Target genes	Molecular mechanism	Biological Functions	Ref.
METTL3	Up	MYC	METTL3-MYC m6A↑-IGF2BP1-MYC↑	Promotes cell proliferation, migration, and invasion	(28)
METTL3	Up	CRB3	METTL3-CRB3 m6A↑-YTHDF2-CRB3↓-MST1, LATS1, MOB1↓; YAP phosphorylation↓-Hippo signaling inactivation	Promotes cell proliferation, migration, and invasion	(29)
METTL3	–	LINC01615	METTL3 - LINC01615 m6A↑-LINC01615↓	Inhibits cell survival during starvation	(30)
METTL3	Up	JAK1	METTL3-JAK1 m6A↑-YTHDF1-JAK1↑-STAT3 phosphorylation↑-JAK/STAT3 signaling activation	Promotes tumor growth and immunosuppression	(31)
METTL3	Up	CCNE1	METTL3-CCNE1 m6A↑-CCNE1↑-Cyclin E1↑	Promotes proliferation, colony formation, and drives cell cycle progression	(32)
METTL3	Up	YPEL5	METTL3-YPEL5 m6A↑-YTHDF2-YPEL5↓- CCNB1, PCNA↑	Promotes proliferation, migration, and metastasis	(33)
METTL3	Up	HK2, GLUT1	METTL3-HK2, GLUT1 m6A↑-IGF2BP2 or IGF2BP2/3-HK2, GLUT1↑-glycolysis signaling activation	Promotes proliferation	(34)
METTL3	–	HMGA1	METTL3-HMGA1 m6A↑- HMGA1↑	Promotes proliferation and metastasis	(35)
METTL3	–	IFIT1	METTL3-IFIT1 m6A↑-IGF2BP2/3-IFIT1↑	Promotes proliferation	(36)
METTL3	Up	SOX2	METTL3-SOX2 m6A↑-IGF2BP2-SOX2↑-CCND1, MYC↑	Promotes CRC cell stemness and metastasis	(37)
METTL3	Up	SOCS2	METTL3-SOCS2m6A↑-SOCS2↓-LGR5↑	Promotes CRC cell stemness and proliferation	(38)
METTL3	–	LBX2-AS1	METTL3-LBX2-AS1 m6A↑-IGF2BP1- LBX2-AS1↑-miR-422a was sponged↑-AKT1↑	Promotes proliferation, migration, invasion, and chemoresistance	(39)
METTL3	–	LINC01559	METTL3- LINC01559 m6A↑-LINC01559↓-miR-106b-5p was sponged↓-PTEN↓	Promotes proliferation and metastasis	(40)
METTL3	Up	HNF1A-AS1	METTL3-HNF1A-AS1 m6A↑-IGF2BP2-HNF1A-AS1↑-miR-93-5p was sponged↑-CCND1↑	Promotes proliferation, migration, and cell cycle progression while inhibits cell apoptosis	(41)
METTL3	–	PTTG3P	METTL3-PTTG3P m6A↑- IGF2BP2-PTTG3P↑-YAP1↑	Promotes proliferation and glycolysis	(42)
METTL3	–	CircQSOX1	METTL3-CircQSOX1 m6A↑-IGF2BP2-CircQSOX1↑-miR-326/miR-330-5p was sponged↑- PGAM1↑	Promotes proliferation, migration, resistance, and immune escape	(43)
METTL3	–	pri-miR-877, pri-miR-483, pri-miR-676	METTL3-pri-miR-877, pri-miR-483, pri-miR-676 m6A↑-miR-877, miR-483, miR-676↑	Promotes proliferation and glycolysis	(44)
METTL3	–	pri-miR-4717	METTL3-pri-miR-4717 m6A↑-miR-4717↑	Promotes proliferation	(45)
METTL3	–	BHLHE41	METTL3-BHLHE41 m6A↑-BHLHE41↑-CXCL1↑	Enhances tumor growth and immunosuppression	(46)
METTL14	Down	SOX4	METTL14-SOX4 m6A↑- YTHDF2-SOX4-PI3K/AKT signaling inactivation	Inhibits migration, invasion, and metastasis	(47)
METTL14	Down	LncRNA XIST	METTL14-lncRNA XIST m6A↑-YTHDF2-XIST↓	Inhibits proliferation and metastasis	(48)
METTL14	Down	Ebi3	METTL14-Ebi3 m6A↑-YTHDF2-Ebi3↓	Inhibits tumor growth	(49)
KIAA1429	Up	HK2	KIAA1429-HK2 m6A↑-HK2↑	Promotes tumor growth	(50)
KIAA1429	Up	SIRT1	KIAA1429-SIRT1 m6A↑-SIRT1↑	Promotes proliferation and migration	(51)

### 2.1 Effects of METTL3 on CRC

MT-A70 is a 70-kDa AdoMet-crosslinked protein, which is also referred to as METTL3 (52). METTL3 is the catalytic subunit in the m6A methyltransferase complex due to its ability to bind with S-adenosylmethionine (SAM) (52). Crucially, the METTL3/METTL14 heterodimers exhibited higher methyltransferase activity than METTL3 or METTL14. METTL3 has weak methyltransferase activity on its own (23, 24). Multiple studies have shown that METTL3 exerts tumor-promoting functions in CRC by regulating gene expression in an m6A-dependent mechanism.

METTL3 has been shown to be highly expressed in CRC tissues and was associated with a poor prognosis in CRC patients (28, 29, 53–56). The expression of METTL3 is upregulated in CRC *via* different mechanisms. Zhang et al. demonstrated that starvation degraded METTL3 through the autophagy–lysosome pathway. More critically, starvation-reduced METTL3 was also reported to upregulate LINC01615 in an m6A modification-dependent manner and then to maintain CRC cell survival (30). A previous study has suggested that METTL3 was SUMOylated by SUMO1, which is responsible for the accumulation of METTL3 in CRC cells (57). Recently, lactate in the tumor microenvironment of CRC has been identified to induce METTL3 expression in tumor-infiltrating myeloid cells *via* H3K18 lactylation. Lactylation on the zinc-finger domain of METTL3 strengthened its capture of m6A-modified RNA (31).

The functional roles of METTL3 in CRC have been investigated *in vitro* and *in vivo*. The knockdown of METTL3 suppressed the proliferation, migration, and invasion of CRC cells, while the overexpression of METTL3 evoked opposite results (28, 29, 32, 33). It is gratifying that the regulatory mechanisms of METTL3 in CRC have been gradually elucidated. Emerging evidence has shown that METTL3 regulates CRC progression through the Hippo pathway. Mechanistically, METTL3 drives m6A modification on CRB3 mRNA in CRC cells; the m6A-YTHDF2 axis represses CRB3 protein translation efficiency and subsequently restrains the activity of Hippo pathways by reducing the level of MST1, LATS1, MOB1, and YAP phosphorylation (29).

A study showed the underlying regulatory mechanism between METTL3-mediated m6A modification and glycolytic metabolism in CRC. Shen et al. revealed that METTL3 acts as a tumor-driver gene and promotes CRC proliferation by accelerating glucose metabolism. METTL3 increased HK2 and SLC2A1(GLUT1) mRNA stability and transcription levels through an m6A-IGF2BP2/3-dependent mechanism, then activated CRC glucose metabolism to facilitate cell proliferation and tumor growth *in vitro* and *in vivo* (34). METTL3 promoted HMGA1 expression in an m6A-dependent manner, which induced tumor cell proliferation and tumor growth in CRC (35).

In addition, METTL3 mediated m6A modification of YPEL5 mRNA and inhibited YPEL5 expression in a YTHDF2-dependent manner, which promoted the proliferation, migration, and metastasis of CRC (33). METTL3 can also enhance the expression of IFIT1 in a way dependent m6A-IGF2BP2/3, resulting in fusobacterium nucleatum-induced PD-L1 upregulation and cell proliferation in CRC (36). Functionally, METTL3 knockdown drastically inhibited CRC stemness *in vitro* and suppressed CRC growth and metastasis in cell-based and PDX-based subcutaneous tumor models. It was proved that METTL3 contributed to an increase in SOX2 transcripts, the key molecule in maintaining the initiating properties of tumor cells, in an m6A-IGF2BP2-dependent mechanism in CRC tumorigenesis (37).

Zhu et al. confirmed that the overexpression of METTL3 stabilized CCNE1 mRNA in an m6A-dependent manner, leading to the proliferation and colony formation of CRC cells. Mechanistically, METTL3 induced the methylation of the m6A site in the 3’-untranslated region (UTR) of CCNE1 mRNA, which stabilized CCNE1 mRNA levels to elevate the protein expression of cyclineE1, thereby exerting a pro-proliferative effect on CRC (32). In addition, research confirmed that overexpression of METTL3 promoted the proliferation, migration, and invasion of CRC cells, as well as induced the cell-cycle progression from the G0/G1 to the S phase.

The knockdown of METTL3 can lead to the opposite effect, yet it has no obvious effect on cell apoptosis. Further studies have found that METTL3 partially showed its function in an m6A-IGF2BP1-dependent manner, which enhanced MYC mRNA stability and transcript levels, therefore promoting MYC gene expression (28). METTL3-mediated m6A modification of SOCS mRNA also facilitates cell proliferation in CRC. Specifically, SOCS2 mRNA was targeted by METTL3. METTL3 enhanced the m6A level of SOCS2 and increased the degradation of SOCS mRNA to exhibit SOCS2 expression. These results contributed to the increased expression of LGR5 and cell proliferation (38). Collectively, the accumulated findings confirmed that METTL3 promoted CRC proliferation and tumorigenesis by regulating a series of mRNAs in an m6A-dependent manner.

A growing body of research shows that METTL3 catalyzes m6A methylation not only by coding RNAs but also by noncoding RNAs in CRC. Ma et al. confirmed the oncogenic role of METTL3 in CRC by inducing lncRNA LBX2-AS1 m6A hypermethylation to enhance its mRNA stability, resulting in significant increases in LBX2-AS1 mRNA in CRC cells. Upregulated LBX2−AS1 increased AKT1 levels by sponging miR−422a, which ultimately led to the proliferation, migration, and 5-fluorouracil resistance of CRC cells (39).

Shi et al. uncovered that LINC01559, a tumor suppressor gene downregulated in CRC, negatively regulated miR-106b-5p expression by interacting with the PTEN axis, thus suppressing CRC proliferation and metastasis. Nevertheless, METTL3 mediated-m6A modification on the LINC01559 restrained the miR-106b-5p/PTEN axis and induced the opposite biological effect. The results verified that METTL3 could bind to and methylate the m6A sites of LINC01559 to affect the functions of CRC cells. Downregulating METTL3 increased the expression of LINC01559 and alleviated CRC cell migration and invasion *in vitro* (40).

A recent study revealed that METTL3 installed the m6A modification and enhanced lncRNA HNF1A-AS1 transcript stability to increase HNF1A-AS1 expression. Upregulated HNF1A-AS1 increased the level of CCND1 by suppressing PDCD4, sponging miR-93-5p and enhancing IGF2BP2-mediated CCND1 mRNA stabilization, which accelerated CRC cell-cycle progression and reduced cell apoptosis (41). Research has found that METTL3 promotes the cell proliferation and glycolysis of CRC by upregulating oncogenic lncRNA PTTG3P. METTL3 was shown to enhance the m6A modification of PTTG3P. IGF2BP2 recognizes and combines with the m6A modification sites of PTTG3P to maintain its stability and expression, which results in YAP1 expression and ultimately promotes the malignant proliferation of CRC cells and CRC progression (42).

Liu et al. confirmed that the m6A modification of circQSOX1 by METTL3 enhanced its expression, depending on the IGF2BP2-dependent pathway. CircQSOX1 sponged miR-326 and miR-330-5p to enhance PGAM1 expression, subsequently activating glycolytic activities to promote proliferation, migration, immune escape as well as tumor resistance (43). It is important that METTL3-dependent m6A modification in pri-miRNAs, such as pri-miR-877, is essential for RALY-mediated mitochondrial metabolism in CRC cells (44). Additionally, METTL3 induces CRC cell proliferation *in vitro* and growth *in vivo* in the context of Fusobacterium nucleatum infection by mediating the m6A modification of pri-miR-4717 and promoting the maturation of miR-4717 (45).

Recent studies have demonstrated that the METTL3-m6A axis inhibits anti-tumor immunity to support CRC growth. Mechanistically, the METTL3-mediated m6A modification of BHLHE41 mRNA promotes the expression of BHLHE41 in an m6A-dependent manner, which subsequently facilitates CXCL1 transcription to recruit myeloid-derived suppressor cells (MDSC). METTL3-driven MDSC accumulation suppresses CD8^+^ T cell proliferation to facilitate CRC growth. More importantly, targeting METTL3 potentiated the effect of anti-PD-1 therapy in suppressing CRC growth (46). Xiong et al. revealed that the increased expression of METTL3 in tumor-infiltrating myeloid cells (TIMs) promotes tumor immune escape in CRC. METTL3 deficiency in TIMs restricted tumor growth and the immunosuppression of TIMs. Further research found that METTL3 promotes the translation efficiency of JAK1 mRNA in the manner of m6A-YTHDF1 and subsequently strengthens STAT3 signaling in TIMs, then drives the immunosuppressive activity of TIMs at tumor sites (31). These findings exhibit a strong pro-tumor response of METTL3-mediated m6A modification in CRC tumorigenesis.

### 2.2 Effects of METTL14 on CRC

In contrast to METTL3, METTL14 was downregulated and represented as an independent risk factor in CRC. In addition, the loss of METTL14 predicted an unfavorable prognosis in CRC patients (47, 48). Chen et al. covered the underlying molecular mechanism of low METTL14 expression in CRC. They found that the KDM5C-mediated demethylation of H3K4me3 in the promoter of METTL14 decreased METTL14 transcription and expression.

In addition, they confirmed that METTL14 inhibited CRC cell migration, invasion, and metastasis. Mechanistically, METTL14 inhibited CRC tumorigenesis by enhancing SOX4 mRNA m6A modification and suppressing the SOX4-mediated EMT process and PI3K/AKT pathway. It is worth noting that METTL14-mediated SOX4 mRNA degradation relied on YTHDF2 (47). Yang et al. identified that METTL14 drastically inhibits the proliferative and metastasis ability of CRC cells. Mechanically, METTL14 targets and inhibits oncogenic lncRNA XIST. It downregulates XIST through an m6A-YTHDF2-dependent pathway. The depletion of METTL14 significantly decreases the m6A levels of XIST and augmented XIST expression. In short, researchers have noticed that METTL14 promotes the m6A methylation of XIST, resulting in its downregulation and CRC progression suppression (48).

Consistent with its anticancer effects in CRC cells, METTL14 in tumor-associated macrophages can also impede CRC growth. Dong et al. discovered that METTL14 depletion in C1q^+^ tumor-associated macrophages promoted the accumulation of Ebi3 mRNA in an m6A-YTHDF2-dependent manner, thereby impairing CD8^+^ T cell infiltration and accelerating CRC growth (49). Thus, METTL14 drove epitranscriptomic regulation in the tumor microenvironment, suggesting paths for the development of potential immunotherapies targeting tumor-associated macrophages.

It is evident that METTL14 from different cells can have different effects on CRC. Notably, the results we have summarized show that METTL14 from tumor cells has a completely opposite effect of METTL3. However, these findings have not been widely validated, and further exploration is needed. The established fact is that METTL3 is the catalytic subunit and its activity strongly depends on METTL14, which is crucial for maintaining complex integrity and recognizing special RNA substrates. From a molecular biology perspective, the opposite roles of METTL3 and METTL14 may be due to their different targets and pathways in RNA methylation. Additionally, certain demethylases may also affect this process. Future research can better understand and explain the different roles of METTL3 and METTL14 in CRC by delving into the interaction between m6A and tumorigenesis.

### 2.3 Other “writers” in CRC

The m6A methyltransferase KIAA1429 acts as an oncogenic factor in colorectal carcinogenesis. Many lines of evidence suggest that KIAA1429 is highly expressed and associated with poor survival in patients with CRC (50, 51, 58). KIAA1429 knockdown substantially inhibited the tumor growth of CRC cells *in vivo via* reducing the m6A level and expression of HK2 mRNA (50). KIAA1429 promoted the proliferation, colony formation, and tumor growth of CRC cells *in vitro* and *in vivo* by downregulating WEE1 expression in an m6A-independent manner or upregulating SIRT1 expression in an m6A-dependent manner (51, 58). Interestingly, butyrate decreased the expression of KIAA1429 by inhibiting the transcription factor NFκB1 (58). Other “writers” are rarely reported in CRC proliferation and tumorigenesis and will not be summarized here.

## 3 The functional roles of m6A “erasers” in CRC proliferation and tumorigenesis

Even though m6A was discovered more than 40 years ago, investigation of its role in gene expression lagged until m6A modification was proven to be reversible through the discovery of FTO as a specific m6A demethylase in 2011 (59). It has been reported that FTO colocalizes with nuclear speckles and exhibits efficient oxidative demethylation activity of abundant m6A residues in RNA, which indicates a reversible regulatory mechanism present in m6A modification (59). ALKBH5, another critical subunit of m6A demethylase, has also been shown to localize in nuclear speckles and is associated with RNA metabolism, mRNA export, and the assembly of the mRNA processing factor, supporting the broad biological roles of the reversible m6A modification on RNA (60). FTO and ALKBH5 belong to the AlkB family of nonheme Fe(II)/α-ketoglutarate (α-KG)-dependent dioxygenases. Accumulating evidence in recent years reveals that FTO and ALKBH5 play key roles in CRC tumorigenesis, depending on their m6A RNA demethylase activity (**Table 2**).

**Table 2:** Functions of m6A “erasers” in CRC.

Gene Symbol	Expression in CRC	Target genes	Molecular mechanism	Biological Functions	Ref.
FTO	Up	MYC	FTO-MYC m6A↓-MYC↑	Promotes proliferation and invasive while inhibits apoptosis	(61)
FTO	Up	SIVA1	FTO-SIVA1 m6A↓-SIVA1↓	Promotes cell growth and colony formation while inhibits apoptosis	(62)
FTO	Up	MZF1	FTO-MZF1 m6A↓-MZF1↑-MYC↑	Promotes proliferation while inhibits apoptosis	(63)
FTO	–	ATF4	FTO-ATF4 m6A↓-YTHDF2-ATF4↑-DDIT4↑-mTOR signaling inactivation	Inhibits tumor cells autophagy while promotes cell survival	(64)
ALKBH5	–	LncRNA NEAT1	ALKBH5-NEAT1 m6A↓-NEAT1↑	Promotes proliferation and migration while inhibits apoptosis	(65)
ALKBH5	Down	PHF20	ALKBH5-PHF20 m6A↓-PHF20↓	Inhibits proliferation and metastasis	(66)
ALKBH5	Down	FOXO3	ALKBH5-FOXO3 m6A↓-FOXO3↑- miR-21↓-SPRY2↑	Inhibits proliferation and metastasis	(67)

### 3.1 Effects of FTO on CRC

FTO was significantly upregulated in CRC tissues and cells (61–63). Lan et al. demonstrated that FTO enhanced CRC cell proliferative and restricted apoptotic ability through the upregulation expression of MYC by removing its m6A modification. The downregulation of FTO resulted in cells arrested at the S phase in addition to the enhancement of apoptotic ability (61). Liu et al. also revealed that FTO depletion suppressed cell growth and colony formation *in vitro* and restricted tumor growth *in vitro* in 5-fluorouracil(5-FU)-resistant CRC cells. Further data confirmed that FTO mediated the m6A demethylation of the apoptotic gene SIVA1 *via* a YTHDF2-dependent mechanism, thereby hindering the apoptotic effect in CRC cells treated with 5-FU (62).

Conversely, increasing FTO expression led to a marked decline in the number of G0/G1-phase cells and an increase in the percentage of S-phase cells, resulting in a decreased proportion of apoptotic cells. Mechanistically, FTO accelerates CRC proliferation through epigenetically promoting m6A/MZF1/c- MYC axis (63). Upregulated glutaminolysis has been discovered in various cancer types and has attracted more attention as a donor of both carbon and nitrogen. It facilitates energy generation and biomass accumulation, providing energy for cancer cell proliferatggion and growth (68). FTO is also involved in the regulation of glutaminolysis inhibition-induced prosurvival autophagy. Han et al. found that FTO is upregulated to stabilize ATF4 mRNA in an m6A-dependent manner by reducing YTHDF2-mediated ATF4 mRNA decay after glutaminolysis inhibition, which induces pro-survival autophagy through suppressing mTOR activity (64).

Upstream signaling regulating the FTO-mediated m6A modification in the CRC cell proliferation was revealed. Research has shown that miR-96 targets AMPKα2 and inhibits its expression in CRC, which subsequently leads to the increased expression of FTO (61). Meanwhile, berberine upregulates FTO expression *via* downregulating β-Catenin (69). GSK3β, a tumor suppressor gene in CRC, mediates the ubiquitination of demethylase FTO to reduce FTO expression, thus hampering the FTO-mediated MZF1/c-MYC axis and inhibiting CRC cell proliferation (63).

### 3.2 Effects of ALKBH5 on CRC

The role of ALKBH5 in CRC progression is not well understood. A study suggested that ALKBH5 acts as a tumor promoter to participate in the development of CRC. Guo et al. confirmed that ALKBH5 enhances cell proliferation and tumor growth while inhibits cell apoptosis in CRC cells. Essentially, ALKBH5 increases the expression level of lncRNA NEAT1 by decreasing its m6A enrichment to promote CRC progression (65). However, ALKBH5 frequently has the opposite effect in CRC. ALKBH5 was significantly downregulated in human CRC tissues and loss of ALKBH5 predicted worse prognosis of CRC patients (66, 67, 70). Functionally, the knockdown of ALKBH5 enhanced the proliferation of colon cancer cells, while the overexpression of ALKBH5 suppressed this ability. Mechanistically, the ALKBH5-mediated m6A modification of PHF20 mRNA inhibited its stability, while the loss of ALKBH5 increased the stability of PHF20 mRNA and prolonged its half-life to facilitate CRC proliferation, colony formation, migration, and invasion (66). In addition, ALKBH5 cooperates with YTHDF3 to facilitate the degradation of circ3823, an oncogene that promotes proliferation and angiogenesis in CRC, and finally inhibits CRC progression. However, it is unknown whether the m6A modification is involved in this process (70). Another study also showed that ALKBH5 plays an antitumor role in CRC: it can inhibit the cell proliferation of CRC by regulating the FOXO3/miR-21/SPRY2 axis in an m6A modification manner (67).

## 4 The functional roles of m6A “readers” in CRC proliferation and tumorigenesis

m6A is installed by m6A methyltransferases, removed by m6A demethylases, and recognized by m6A-binding proteins. Its m6A-binding proteins regulate RNA metabolism, including translation, splicing, export, degradation, and microRNA processing. m6A-binding proteins contain the YT521-B homology (YTH) domain family, insulin-like growth factor 2 mRNA-binding proteins (IGF2BPs), and heterogeneous nuclear ribonucleoproteins (HNRNPs) (71–73); Accumulating evidence suggests that m6A “readers” are greatly implicated in many human cancers, such as CRC (**Table 3**).

**Table 3:** Functions of m6A “readers” in CRC.

Gene Symbol	Expression in CRC	Target genes	Molecular mechanism	Biological Functions	Ref.
YTHDF1	Up	ARHGEF2	YTHDF1-ARHGEF2 m6A-ARHGEF2↑-RhoA signaling activation	Promotes tumor growth, migration, invasion, and metastasis	(6)
YTHDF1	Up	TCF7L2, TCF4	YTHDF1-TCF7L2, TCF4 m6A-TCF7L2, TCF4↑-Wnt/β-catenin signaling activation	Promotes stemness and tumor growth	(74)
YTHDF1	Up	GMEB2	GMEB2 m6A-YTHDF1- GMEB2↑-ADRM1↑-NF-κB signaling activation	Promotes proliferation	(75)
YTHDF1	Up	SH3TC2	SH3TC2 m6A-YTHDF1- SH3TC2↑	Promotes proliferation	(76)
YTHDF2	Up	GSK3β	YTHDF2-GSK3β m6A- GSK3β↓-Wnt/β-catenin signaling activation-Cyclin D1↑	Promotes cell cycle progression and proliferation	(77)
YTHDF2	–	Circ 0003215	YTHDF2-circ 0003215 m6A-circ 0003215↓	promotes proliferation, invasion and migration	(78)
YTHDF2	–	STEAP3	YTHDF2-STEAP3 m6A-STEAP3↓-Wnt/β-catenin signaling inactivation	Inhibits proliferation and metastasis	(83)
YTHDF2	–	DEGS2	DEGS2 m6A- YTHDF2- DEGS2↓	Inhibits proliferation and metastasis	(79)
YTHDF3	Up	LncRNA GAS5	YTHDF3-GAS5 m6A-GAS5↓	Promotes proliferation and invasion	(80)
YTHDC1	Down	circFNDC3B	YTHDC1- circFNDC3B m6A- circFNDC3B↑	Inhibits stemness and metastasis	(81)
IGF2BP2	Up	MYC	IGF2BP2- MYC m6A-MYC↑-GLUT-1, PKM2, LDHA↑	Promotes proliferation	(82)
IGF2BP2	Up	CREB1	IGF2BP2- CREB1 m6A- CREB1↑	Promotes proliferation and migration	(83)
IGF2BP2	Up	ZFAS1	IGF2BP2-ZFAS1m6A-ZFAS1↑-OLA1 activity↑- Warburg effect activation	Promotes proliferation while inhibits apoptosis	(84)
IGF2BP2	Up	YAP	IGF2BP2- YAP m6A- YAP↑- ERBB2↑	Promotes proliferation, migration, and invasion while inhibits apoptosis	(85)
IGF2BP2	–	MSX1, JARID2	IGF2BP2- MSX1, JARID2 m6A- MSX1, JARID2↑	Promotes proliferation, migration, and invasion while inhibits apoptosis	(86)
IGF2BP3	Up	CCND1	IGF2BP3-CCND1 m6A-CCND1 mRNA↑-Cyclin D1↑	Promotes proliferation and cell cycle progression	(53)
FMR1	Up	EGFR	FMR1- EGFR m6A- EGFR↑	Promotes cell cycle progression and metastasis while inhibits apoptosis	(87)

### 4.1 Effects of YTHDFs on CRC

YTH domain-containing proteins include YTH N6-methyladenosine RNA-binding proteins (YTHDFs) and YTH domain-containing (YTHDCs) families. There are five human YTH domain-containing proteins: YTHDF1, YTHDF2, YTHDF3, YTHDC1, and YTHDC2. They are distributed in the nucleus and cytoplasm, bind to m6A, and influence the fate of m6A-containing RNA in mammalian cells, such as through RNA metabolism, RNA folding, RNA splicing, and protein translation (88, 89). Numerous studies have demonstrated the roles of YTHDFs in CRC proliferation and tumorigenesis.

YTHDF1 actively promotes the mRNA translation and protein synthesis of m6A-modified mRNA (18). In contrast to the mRNA stability-promoting function of YTHDF1, YTHDF2 induces the decay of its target transcripts in an m6A-depedent manner (19). The biological function of YTHDF3 is to promote protein synthesis in synergy with YTHDF1 and to enhance methylated mRNA decay mediated through YTHDF2 (20). The participation of YTHDCs in the development of CRC proliferation has not yet been explored.

YTHDF1 was significantly increased in CRC tissues compared with adjacent non-tumor tissues, and the copy number gain/amplification of YTHDF1 contributes to its upregulation in CRC (6, 90). Intriguingly, YTHDF1 was targeted and inhibited *via* miR-136-5p in CRC cells. However, circPTK2 enhanced YTHDF1 expression by competitively binding to miR-136-5p (90). YTHDF1 was found to be highly expressed in human CRC tissues and predominantly localized in intestinal stem cells. Its expression is upregulated by Wnt3a and downregulated by APC at the translational level. Meanwhile, it is required for WNT-driven intestinal stem cell regeneration and intestinal tumor development, as YTHDF1 can facilitate the translation of TCF7L2/TCF4, the major transcriptional effector for β-catenin in mouse intestine and human colorectal cancer cells (74).

Functionally, YTHDF1 promotes the cell proliferation and metastasis capacity of CRC cells and the growth of primary CRC organoids. The YTHDF1 knockdown showed a reduction in tumor number and an inhibition of tumor growth in the AOM/DSS-induced CRC mouse model (6, 74). Noticeably, YTHDF1 binds to the m6A sites of ARHGEF2 mRNA, resulting in enhanced translation of ARHGEF2 and CRC tumorigenesis (6). A recent study uncovered that the GMEB2/ADRM1 axis promoted CRC growth by activating NF-κB signaling. This process was enlarged by YTHDF1 due to its ability to increase the stability of GMEB2 mRNA in an m6A-dependent way (75). In addition, YTHDF1 can directly bind with SH3TC2 mRNA and promote its upregulation in an m6A-dependent manner, then facilitate cell-cycle progress and growth in CRC (76). It follows then that YTHDF1 acts as an oncogenic factor in colorectal tumorigenesis.

YTHDF2 was dramatically upregulated in CRC tissues compared with adjacent normal tissues (77, 91). Most studies suggest that YTHDF2 functions as an oncogene in CRC cells. YTHDF2 overexpression significantly promoted the proliferation of CRC cells. Further mechanism analysis demonstrated that YTHDF2 inhibited the expression of GSK3β *via* decreasing the stability of GSK3β mRNA. Decreased GSK3β protein levels increased the expression of WNT/β-catenin/Cyclin D1 pathway-related proteins, leading to cell-cycle progression from G0 to G1 and ultimately promoting the proliferation of CRC cells. Intriguingly, YTHDF2 was the direct target gene of miR-6125 in CRC cells, and it was downregulated and served as a suppressor gene in CRC. Its mRNA translation was inhibited by miR-6125 (77). Recently, the tumor-suppressing function of circ0003215 in CRC proliferation, invasion, and migration has been demonstrated. Importantly, YTHDF2 promotes the RNA degradation of circ0003215 (78). The above studies highlight the important roles of YTHDF2 in promoting CRC proliferation and tumorigenesis. However, YTHDF2 has also been shown to inhibit CRC. Zhou et al. revealed that hypoxia-induced LncRNA STEAP3-AS1 facilitates the proliferation and metastasis of CRC cells both *in vitro* and *in vivo*. YTHDF2 is a critical molecular involved in the above-mentioned malignant biological behavior by negatively regulating the expression of STEAP3. STEAP3-AS1 competitively binds to YTHDF2, leading to the disassociation of YTHDF2 with STEAP3 mRNA and then preventing the m6A-mediated degradation of STEAP3 mRNA, thus activating Wnt/β-catenin signaling to support CRC progression (92). Researchers verified that YTHDF2 is involved in the regulation of DEGS2 by mediating the degradation of m6A-modified DEGS2 mRNA, resulting in the inhibition of CRC proliferation and migration (79) It appears that YTHDF2 may act as a double-edged sword in CRC tumorigenesis.

YTHDF3 facilitates protein synthesis in synergy with YTHDF1 and affects methylated mRNA decay mediated through YTHDF2 (20). It was observed that the expression of YTHDF3 was higher in CRC tissues than in normal mucosal tissues. The higher expression of the YTHDF3 protein was a significant prognostic factor for poor overall survival in CRC patients. Increased YTHDF3 binds to m6A-methylated lncRNA GAS5 to trigger its decay and then inhibits YAP phosphorylation to restrain YAP ubiquitination and degradation, which promote CRC cell proliferation and invasion. Surprisingly, YTHDF3 not only a key player in YAP signaling but also a novel target of YAP. Research found that there is a negative functional loop of the lncRNA GAS5-YAP-YTHDF3 axis during the progression of CRC. The overexpression of YAP increased YTHDF3 expression, while the knockdown of YAP showed the opposite results (80). However, a recent report presented a different view on the function of YTHDF3 in CRC. Guo et al. showed that YTHDF3 inhibits CRC growth, metastasis, and angiogenesis by promoting the degradation of m6A-modified circ3823 (70). Therefore, the same YTHDF3-mediated m6A machinery has two sides of the same coin. This may lead to either pro- or anti-proliferation effects, while the underlying mechanism demands further study.

### 4.2 Effects of YTHDCs on CRC

YTHC1 and YTHDC2 are YTH domain-containing proteins found in the mammalian genome that confer m6A-dependent RNA-binding activity. Studies have implicated the multifaceted effects of YTHDC1-mediated nuclear m6A recognition, such as alternative splicing and mRNA export (93–95). Compared to normal bowel tissues, YTHDC1 was downregulated in colon cancer (53). A recent study demonstrated that YTHDC1 accelerated the cytoplasmic translocation of m6A-modified circFNDC3B, thereby inhibiting CRC stemness and metastasis *via* RNF41-dependent ASB6 degradation (81).

YTHDC2 has been suggested to play multiple roles, including mediating mRNA stability and translation and regulating spermatogenesis (96). Analysis of the TCGA dataset revealed that YTHDC2 was downregulated in human colonic adenocarcinoma tissues compared to normal tissues. Additionally, YTHDC2 exhibited a downward trend as clinical stage increased (53, 97). Liu et al. also observed a significant decrease in YTHDC2 in CRC tissues. They further indicated that lower expression of YTHDC2 is an independent, worse prognostic factor in CRC patients (98). However, the additional functions of YTHDC2 in CRC remain enigmatic. Overall, the reported effects are small, which raises the possibility that the function of YTHDCs in CRC has not been fully elucidated.

### 4.3 Effects of IGF2BPs on CRC

IGF2BPs, including IGF2BP1, IGF2BP2, and IGF2BP3, as distinct and conserved m6A “readers,” strengthen the stability and translation efficiency of m6A-modified mRNAs and thus affect gene expression output through recognizing the consensus GG (m6A) C sequence. They are required in the post-transcriptional gene regulation and cancer biology of CRC (99, 100).

Several studies have confirmed that IGF2BP2 was highly expressed in CRC tissues. Higher expression of IGF2BP2 led to significantly poorer overall survival in CRC (82–84). A recent work revealed a novel regulation mechanism by the IGF2BP2/ZFAS1/OLA1 signal axis in CRC proliferation. Functionally, IGF2BP2 promoted CRC cell proliferation and inhibited CRC cell apoptosis by promoting ZFAS1 stability and expression. Lu et al. confirmed that IGF2BP2 directly binds to the m6A site on ZFAS1 through KH3-4, enhancing its stability and expression in an m6A dependent manner. Notably, the IGF2BP2 stabilized ZFAS1 subsequently enhanced OLA1 activity and activated glycolysis by binding to the OBG-type functional domain of OLA1, which enhanced the ATPase activity of the OLA1 protein and activated the Warburg effect, ultimately promoting CRC cell proliferation (84). IGF2BP2 has been found to promote the progression of CRC through a YAP-dependent mechanism. Concretely, IGF2BP2 bound to m6A-modified YAP mRNA and then increased its stability in CRC cells, resulting in the increased expression of ERBB2. Then the cell proliferation was promoted, and apoptosis was repressed (85). In addition, IGF2BP2 exerted its function as an mRNA-stabilizing RNA-binding protein through recognizing the m6A-modified element RGGAC of MSX1 and JARID2, thereby promoting the cell proliferation and growth of CRC (86).

Interestingly, the expression and biological behaviors of IGF2BP2 were regulated by non-coding RNA in CRC. Research revealed that lncRNA LINRIS maintained the stability of IGF2BP2 by suppressing its ubiquitination by masking K139. This process was realized through the ubiquitination–autophagy pathway. The interaction between LINRIS and IGF2BP2 effectively influenced MYC-mediated proliferation and growth in CRC (82). circEZH2 was reported to interact with IGF2BP2 and block its ubiquitination-dependent degradation. Meanwhile, circEZH2 acts as a sponge of miR-133b, leading to the upregulation of IGF2BP2. Mechanistically, circEZH2/miR-133b/IGF2BP2 facilitates the proliferation of CRC cells *via* enhancing the stability of m6A-modified CREB1 mRNA (83). LINC00460, IGF2BP2, and DHX9 have been shown to interact with each other to promote CRC proliferation and metastasis by mediating HMGA1 mRNA stability, depending on m6A modification (35). In addition, LINC021 directly binds to the “CCCAC” fragment of the IGF2BP2 protein, which enhanced the IGF2BP2-dependent stabilization of MSX1 and JARID2 and induced cell proliferation, colony formation, and cell-cycle progression in an m6A regulatory manner (86).

IGF2BP3 is more highly expressed in CRC than in normal tissues. Patients with high IGF2BP3 expression have significantly poorer overall survival (53, 101). Yang et al. demonstrated that the knockdown of IGF2BP3 significantly inhibited CRC proliferation by repressing the percentage of the S phase of the cell cycle but promoting cell cycle arrest in the G0/G1 phase. Subsequently, they confirmed that IGF2BP3 regulated the CRC cell cycle and proliferation by reading m6A modifications in CCND1 and promoting its mRNA expression. The knockdown of IGF2BP3 significantly decreased the stability and halftime of CCND1 mRNA as well as the protein expression of Cyclin D1, which inhibits the cell cycle and DNA replication (53).

### 4.4 Other “readers” in CRC

The biological functions of hnRNPA2B1 in CRC have recently been revealed. Studies have shown that hnRNPA2B1 promotes colon cancer cell proliferation, and the knockout of hnRNPA2B1 significantly induces apoptosis and cell cycle arrest. The underlying mechanism shows that hnRNPA2B1 promotes cell proliferation and inhibits cell apoptosis of CRC cells by activating the ERK/MAPK signaling (102). However, whether hnRNPA2B1 promotes CRC cell proliferation in an m6A-dependent manner or not has not been elucidated. FMR1, a new m6A reader, is known to recognize the m6A-modification site in EGFR mRNA, sustain its stability, and maintain its expression in an m6A-dependent manner, thereby prohibiting apoptosis and promoting the cell-cycle progression of CRC cells (87).

As is commonly known, reader proteins such as HNRNPC and HNRNPG act as “m6A-switches” that regulate primary miRNA processing, pre-mRNA alternative splicing, and processing in multiple types of tumors (73). However, their effects on CRC proliferation and tumorigenesis need to be explored and verified in the future in depth.

## 5 Potential therapeutic implications of m6A modification in tumors

The search for a connection between epigenetics and human cancer has a long history. Major advances have been made in understanding colorectal cancer epigenetics, particularly regarding aberrant DNA methylation, histone modification, and non-coding RNAs (103). These advances in CRC have led to epigenetic alterations being developed as therapeutic implications (103). Epigenetic alterations are essentially reversible, which makes them attractive therapeutic targets in a variety of diseases. The two classic epigenetic modifying drugs are DNA methyltransferase (DNMT) inhibitors and histone deacetylase (HDAC) inhibitors, some of which have been tested preclinically or in early-phase clinical trials in CRC. For example, the DNMT inhibitors decitabine, azacytidine, and zebularine showed synergic effects with anticancer drugs such as 5-FU, irinotecan, and oxaliplatin. In addition, the HDAC inhibitor vorinostat or LBH589 was shown to overcome, at least partially, thymidylate synthase-mediated 5-FU resistance by enhancing cell cycle arrest and inhibiting growth in colon cancer cells (104).

**Figure 2:**
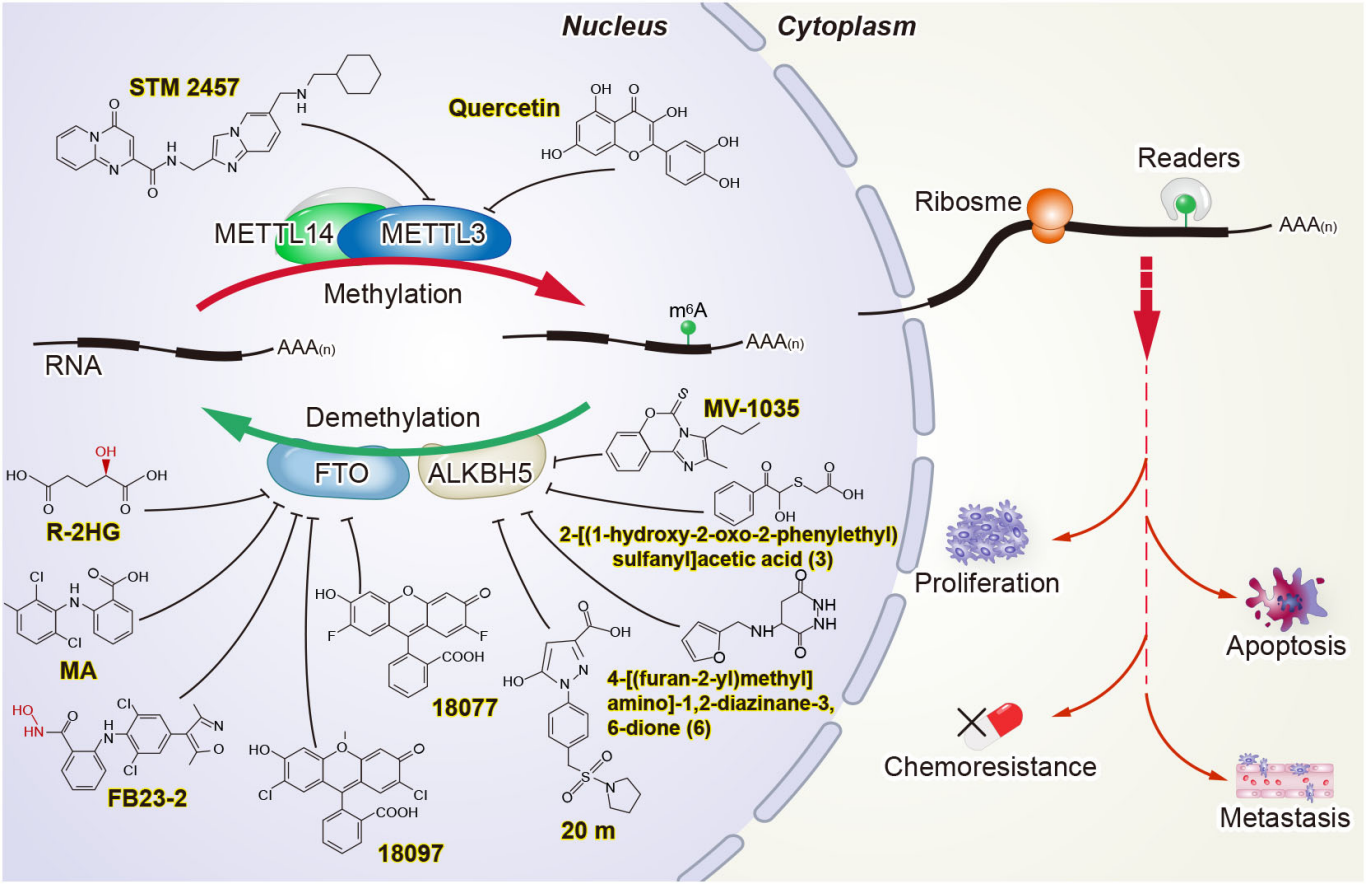
Potential strategies for targeting m6A in cancer.

m6A levels were found to be substantially elevated in CRC and were associated with poor prognosis (29, 53). There is no doubt that m6A modification is critical for tumorigenesis and aggressiveness in CRC. More importantly, m6A modification holds promise for the future therapeutics of CRC (**Figure 2**).

### 5.1 Inhibitors of “Writers”

Research on m6A in cancer has not only provided attractive biomarker candidates but has also opened the door for the development of new anticancer drugs (105–109). “Writers” and “erasers” are promising therapeutic targets, since their activity can be modulated by small molecules. STM2457 was identified as a highly potent and selective catalytic inhibitor of METTL3, with a IC_50_ of 16.9 nM. Yankova et al. verified that STM2457 can directly bind to the SAM binding site of METTL3, thereby inhibiting the methyltransferase activity of METTL3. Treatment with STM2457 leads to reduced acute myeloid leukemia cell growth and an increase in differentiation and apoptosis (110). Du et al. found quercetin to be a small molecule inhibitor of METTL3 through the virtual screening of natural products. Furthermore, molecular docking studies revealed that quercetin efficiently binds to the pocket of the adenosine moiety of SAM in the METTL3 protein and forms a stable protein-ligand complex. Quercetin decreased m6A levels and inhibited proliferation in a dose-dependent manner in human pancreatic cancer cells and hepatocellular carcinoma cells (111). Several potentially small-molecule inhibitors of METTL3 have been discovered by a cofactor mimicking approach (112), while the promising inhibitors have some limitations, and verification is needed in the future.

### 5.2 Inhibitors of “erasers”

Other studies have attempted to develop small-molecule inhibitors to target m6A “erasers.” For example, FTO inhibitors have been developed by biochemical- or cell-based small-molecule compound library screening or chemical synthesis. FTO was verified to be inhibited by R-2-hydroxyglutarate (R-2HG), thereby increasing m6A RNA modification in R-2HG-sensitive leukemia cells and decreasing the stability of MYC/CEBPA transcripts, which leads to the suppression of leukemia cell proliferation/viability and the inhibition of cell-cycle arrest and apoptosis (113). FB23 and FB23-2 are promising FTO inhibitors. Both can directly bind to FTO and selectively inhibit FTO’s m6A demethylase activity. At the same time, FB23-2 dramatically restrains proliferation and promotes the differentiation or apoptosis of acute myeloid leukemia cells *in vitro* and *in vivo* (114). In addition, two novel FTO inhibitors, namely 18077 and 18097, were recently identified by Xie et al. They showed that 18097 bound to the active site of FTO and inhibited the cell cycle process and migration of cancer cells (115). Meclofenamic acid, a non-steroidal anti-inflammatory drug, was also found to be a highly selective inhibitor of FTO over ALKBH5 (116).

Compound 20 m was reported as a potent, selective, and cell-active ALKBH5 inhibitor containing the 1-aryl-1H-pyrazole scaffold (117). The imidazobenzoxazin-5-thione MV1035 was tested to inhibit ALKBH5 demethylase activity and to reduce glioblastoma cell migration and invasiveness *in vitro* (118). In addition, two ALKBH5 inhibitors, 2-[(1-hydroxy-2-oxo-2-phenylethyl)sulfanyl]acetic acid (3) and 4-[(furan-2-yl)methyl]amino]-1,2-diazinane-3,6-dione (6), have been shown to suppress proliferation in leukemia cell lines *in vitro* (119).

Although there are currently no small molecule inhibitors of “reader” proteins, the aberrant expression and function of “readers” in CRC might provide the impetus for the development of inhibitors to them. A recent study has reported that silence c-Myc inhibited YTHDF1 expression, resulting in the suppression of colorectal cancer proliferation and the sensitization to the exposure of anticancer drugs, such as 5-FU and oxaliplatin (120). However, more awareness of RBPs’ crystal structure needs to be well documented, and inhibitors require further discovery or development.

These findings represent an important step toward the development of m6A methyltransferase and demethylase inhibitors, although the effects of these inhibitors have not been validated in colorectal cancer.

## 6 Conclusions and perspectives

The dynamic regulation of m6A maintains the balance of physiological and pathological processes. m6A regulates numerous biological processes that are essential to the genesis of cancer, including CRC. A deeper understanding of epigenetic regulatory mechanisms in CRC will facilitate the exploration of future clinical applications.

In this review, we have summarized the roles of several key m6A proteins in CRC proliferation and tumorigenesis that have been studied to date, some of which have promising therapeutic implications, including METTL3, FTO, and ALKBH5. From the collected evidence, it can be concluded that some of the “writers”, “erasers”, and “readers” are potential biomarkers for the diagnosis of CRC. Despite significant progress in recent years, the exact mechanism of m6A modification remains incomplete and elusive. Our understanding of how m6A modification contributes to CRC is still in its infancy.

Given the complexity of CRC tumorigenesis, it is important to also consider other cancer-related factors, including diet and nutrition, obesity, and the intestinal microbiome, in the regulation of m6A. Furthermore, the analysis of tissue-specific knock-out mice of “writers”, “erasers”, or “readers” can help elucidate the divergent intracellular biological functions. Further clinical trials are necessary to assess the potential diagnostic and therapeutic effects of m6A modification in CRC patients. As m6A mapping approaches and m6A editing tools are developed, much fundamental work will be accomplished. Once these problems are solved, it may be possible to design specific small molecules that target m6A modification. Moreover, the results of basic research on m6A modification in CRC will soon be tested in clinical trials and may eventually be applied in clinical practice.

## Author contributions

ZJ wrote the first draft. XJ wrote part of the manuscript and contributed to the graphic production. XMZ and NQ contributed to the concept and design of the review. YY contributed to the graphic design. XZ, SC, SW comments on the revision of the review are provided. All authors listed have made a substantial, direct, and intellectual contribution to the work and approved it for publication.
